# Valorization of Algal Biomass to Produce Microbial Polyhydroxyalkanoates: Recent Updates, Challenges, and Perspectives

**DOI:** 10.3390/polym16152227

**Published:** 2024-08-05

**Authors:** Anand Narayanasamy, Sanjay K. S. Patel, Neha Singh, M. V. Rohit, Jung-Kul Lee

**Affiliations:** 1 Bioconversion Technology Division, Sardar Patel Renewable Energy Research Institute, Vallabh Vidyanagar, Anand 388120, Gujarat, India; narayanasamy.anand@gmail.com (A.N.); bio4@spreri.org (N.S.); bio3@spreri.org (M.V.R.); 2 Department of Biotechnology, Hemvati Nandan Bahuguna Garhwal University (A Central University), Srinagar 246174, Uttarakhand, India; sanjaykspatel@hnbgu.ac.in; 3 Department of Chemical Engineering, Konkuk University, 120 Neungdong-ro, Gwangjin-gu, Seoul 05029, Republic of Korea

**Keywords:** algal biomass, biodegradable plastics, environmental pollution, polyhydroxyalkanoates, reducing sugars

## Abstract

Biopolymers are highly desirable alternatives to petrochemical-based plastics owing to their biodegradable nature. The production of bioplastics, such as polyhydroxyalkanoates (PHAs), has been widely reported using various bacterial cultures with substrates ranging from pure to biowaste-derived sugars. However, large-scale production and economic feasibility are major limiting factors. Now, using algal biomass for PHA production offers a potential solution to these challenges with a significant environmental benefit. Algae, with their unique ability to utilize carbon dioxide as a greenhouse gas (GHG) and wastewater as feed for growth, can produce value-added products in the process and, thereby, play a crucial role in promoting environmental sustainability. The sugar recovery efficiency from algal biomass is highly variable depending on pretreatment procedures due to inherent compositional variability among their cell walls. Additionally, the yields, composition, and properties of synthesized PHA vary significantly among various microbial PHA producers from algal-derived sugars. Therefore, the microalgal biomass pretreatments and synthesis of PHA copolymers still require considerable investigation to develop an efficient commercial-scale process. This review provides an overview of the microbial potential for PHA production from algal biomass and discusses strategies to enhance PHA production and its properties, focusing on managing GHGs and promoting a sustainable future.

## 1. Introduction

Plastics are polymers widely used in various human activities because of their exceptional physicochemical properties, affordability, and practicality [1,2]. However, plastics pose significant environmental hazards attributed to their slow degradation under normal circumstances, resulting in their accumulation in nature [3,4]. To address this issue, research is underway to develop biodegradable plastics [5,6,7]. Among the different biopolymers produced metabolically by various microorganisms, polyhydroxyalkanoates (PHAs) have emerged as promising alternatives to plastics [8,9]. PHAs are biodegradable; however, they are produced in only limited amounts by certain prokaryotes and eukaryotes [10]. Specific microorganisms can synthesize poly-β-hydroxybutyrates (PHBs), a class of PHAs. However, the fragile nature and poor physicochemical properties of PHBs limit their scope for biotechnological applications and, thus, their commercialization [11,12]. Additionally, the cost of this bioprocess approach is affected by feed costs, which can account for up to 45% of the total cost [9,10]. These restrictions have resulted in a search for biowaste as an alternative feedstock for PHA production [12,13].

Microbial fermentation of biowaste is more challenging than that of pure sugars because of its complex nature and diverse composition. Therefore, pretreatment is required for better accessibility to enzymatic/microbial hydrolysis, facilitating the production of fermentable sugars or bioactives [14,15,16]. Biological methods are generally helpful, owing to the clean transformation of biowaste into biofuels, biopolymers, or other value-added products [9,17,18,19]. PHAs are aliphatic polyesters produced by numerous prokaryotic organisms, which can constitute up to 90% of their dry cell weight (DCW) [10]. Fundamentally, the synthesis of PHAs occurs under physiological stress conditions, including an abundance of carbon (C) sources in the growth environment and lower concentrations of essential elements, such as magnesium, potassium, nitrogen (N), phosphorus (P), sulfur, or iron, during their development period [10,20]. PHAs can be blended into either homopolymer or copolymer structures. PHB is the most popular homopolymer of PHA and is produced by many microbes. The biosynthesis of PHB involves three crucial steps: (i) the initial step is catalyzed by β-ketothiolase (encoded by *phaA*, which involves the condensation of two acetyl-CoA molecules to form acetoacetyl-CoA); (ii) the acetoacetyl-CoA is reduced to acetoacetyl-CoA by NADPH-dependent acetoacetyl-CoA dehydrogenase (encoded by *phaB*); (iii) finally, the monomers [(R)- 3-hydroxybutyryl-CoA] are polymerized into PHB by PHB synthase (encoded by *phaC*) [9,21]. The type of PHB produced by various organisms depends on the *phaC* present in specific organisms [10,11].

Microorganisms, including Gram-positive bacteria such as *Bacillus*, *Rhodococcus*, and *Staphylococcus*; Gram-negative bacteria such as *Acinetobacter*, *Azotobactor*, *Burkholderia*, *Halomonas*, *Klebsiella*, *Pseudomonas*, and *Ralstonia*; algae such as *Arthrospira*, *Botryococcus*, *Chlamydomonas*, *Chlorella*, *Nostoc*, and *Spirulina*; and engineered microorganisms such as *Escherichia*, *Halomonas*, *Bacillus*, and *Saccharomyces* have been reported for PHA production [10,22,23,24]. Microbial PHA production via fermentation has been widely reported using—(i) pure sugars such as glucose, fructose, maltose, and starch and (ii) biowastes of diverse origins, including agricultural (rice straw, wheat straw, potato, onion, carrot, cauliflower, tomato, pea-shells, orange peels, grape peels, melon, and apple pulp), municipal (vegetable, fruit, and other food waste), industrial (algal biomass, molasses, cheese whey, biodiesel waste, and dairy waste), and synthetic (wastewater) sources [9,23,25]. Microbial PHAs are classified into two significant subdivisions based on the C chain length of their monomeric units: short-chain-length (scl) PHAs with 3–5 C atoms and medium-chain-length (mcl) PHAs with 6–18 C atoms [26,27].

Microalgae are photosynthetic microorganisms capable of converting carbon dioxide (CO_2_) and sunlight into biomass [23,28]. They have faster growth rates and do not compete with food crops for arable land or freshwater resources. Additionally, microalgae can be cultivated using waste streams such as CO_2_ derived from flue gas and wastewater, further improving the sustainability of the production process [29,30]. In estuarine environments, benthic macroalgae can account for a significant portion of total primary production, sometimes up to 50% [29]. The chemical composition of biomass, including carbohydrates (7.8–30.8%), proteins (13.0–65.2%), and lipids (3.2–30.4%), is quite variable in red, brown, and green algae along with various pigments and secondary metabolites [23,28]. The simple structure of microalgae, which contains less lignin than other renewable biomass, can facilitate the bioprocessing of their carbohydrate fraction for producing PHAs. Carbohydrate recovery from algal biomass highly depends on pretreatment methods, such as biological, physical, and chemical methods, which significantly vary among types of algae due to their diverse composition [31,32]. Algae are widely preferred for the bioremediation of toxic compounds from the wastewater of diverse origins and are also effective in mitigating greenhouse gases (GHGs) such as CO_2_. The production of algal biomass using wastewater or CO_2_ for biomass production, followed by the further utilization of biomass hydrolysate to produce PHAs, presents a sustainable approach to achieving a circular economy [13,33,34]. Moreover, microbes such as *Bacillus*, *Cupriavidus*, *Halomonas*, *Haloferax*, *Paracoccus*, and *Rhodotorula* demonstrate high potential for producing PHAs with yields up to 22.5 g/L from algal-biomass-derived sugars [23,35,36,37]. The microbial production yield and composition of PHAs from algal biomass are highly dependent on factors such as pH, temperature, incubation period, the type of limitation (such as N- and P-sources), the origin of feed, and the kind of PHA-producing culture [31,36,38,39,40]. Monomeric forms of PHAs, such as PHB, are widely produced by these microbes using algal biomass [35,36,38,39]. A few studies have reported the microbial production of PHA copolymers as poly-3-hydroxybutyrate-*co*-3-hydroxyvalerate [P(3HB-*co*-3HV)] from algal-derived sugars or residues [37,41,42]. Generally, copolymer precursors such as valerate are required for the microbial synthesis of P(3HB-*co*-3HV) using pure or lignocellulosic-biomass-derived sugars [4,43]. In contrast, *Halomonas mediterranei* DSM 1411 can efficiently produce P(3HB-*co*-3HV) copolymers lacking any external precursor addition [38]. Additionally, the properties of the produced PHAs must be suitable for biotechnological applications, particularly in tissue engineering and drug carrier biomedical applications [3,43]. Largely produced PHB by microbes using sugars or algal biomass demonstrate low potential for biomedical applications. Given the broad benefits of PHA copolymers, it is highly recommended that they be easily synthesized by various methods, such as altering the feed sources, production conditions, and the use of engineered microbes [23,44,45,46]. Therefore, different technologies require the integration of complementary microorganisms and algal biomass as feed to produce various PHA copolymers with diverse properties. The recent pandemic has highlighted the need for waste management approaches and technological advancements for sustainable development. This review aims to evaluate the existing literature on microbial PHA production from algal-based biomass hydrolysates. Furthermore, various strategies and challenges have been discussed to improve PHA production and develop sustainable approaches that offer environmental benefits.

## 2. Algal Biomass and Their Valorization Approaches

**Figure 1:**
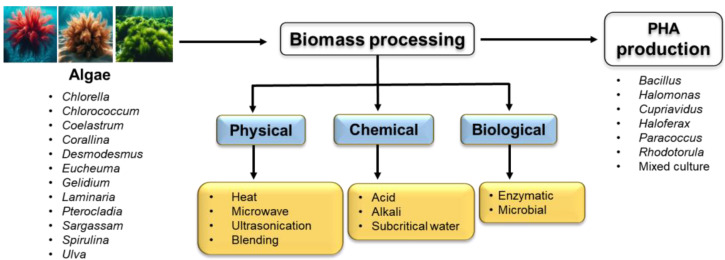
Process flow for the microbial conversion of algal biomass to PHAs.

Pure sugars are the preferred substrates for biotransformation applications. However, their high cost limits large-scale conversion, owing to their low economic feasibility. Therefore, inexpensive feedstocks such as sugars derived from biowaste(s) are being explored to develop an efficient biotransformation system on a pilot scale [14,18,47]. The lignocellulosic composition of biomass, comprising cellulose, hemicellulose, and lignin, varies significantly depending on the source of biomass, such as plants or algae [48,49,50]. The high contents of lignin, up to 22% in woody biomass, result in a less efficient system feedstock due to the partial release of fermentable sugars in hydrolysate or the production of undesirable byproducts inhibiting biotransformation approaches or microbial fermentation [9]. Various pretreatment approaches, such as physical, chemical, and biological approaches, have been widely studied; however, optimal pretreatment approaches or their combinations are under consideration to maximize the extraction of sugars from biomass while limiting inhibitory components, such as phenolics [51,52,53]. Algal-derived biomass is highly desirable for producing fermentable sugars compared to plant-based biomass because of its low lignin content (of up to 5%) [54]. In general, microalgal biomass is rich in various proteins (10–47 wt% DCW), starch components (10–20 wt% DCW), amylopectin (80–90 wt% DCW), cellulose, and lipids (20–50 wt% DCW) [55,56,57]. The high lignin content in woody biomass, especially rice straw, wheat straw, and agro-residues, yielded lower PHAs compared to microalgal biomass. Of the various strategies involved in the pretreatment of microalgal biomass, acid and enzymatic hydrolysis are the most commonly used saccharification methods to convert biomass into reducing sugars [58,59,60,61]. Other pre-treatment methods, especially physical pretreatment methods, can aid in extracting intracellular byproducts from microalgae; however, they cannot be used to produce the reducing sugars required for PHA production [13,53,62,63]. Primarily, microalgal biomass, including its hydrolysates, often contains toxic inhibitory compounds such as phenolics, which can significantly reduce microbial growth and fermentative product yields, including PHA accumulation [23]. Therefore, hydrolysate detoxification is often necessary before fermentation [13,64,65,66]. The bioprocess approaches involved in PHA production using algal biomass are presented in Figure 1.

Wang et al. [67] reported achieving a high reducing sugar yield of up to 90% at an enzyme loading of cellulase (11.5 U/mL) and amylase (304 U/mL) from sulfuric acid pretreated *Chlorella vulgaris* JSC-6 biomass (120 g/L). Enzymatic hydrolysis using a combination of cellulase and amylase yielded up to 0.44 g/g, reducing the sugar content. Castro et al. [68] achieved a lower reducing sugar yield (0.17 g/g during acid hydrolysis of mixed microalgal biomass (dominated by *Ankistrosdemus*, *Chlamydomonas*, *Chlorella*, *Micromonas*, and *Scenedesmus*)). This hydrolysis process is species-specific, and the yield of reducing sugars varies significantly depending on the type of microalgal species. Furthermore, the suitable ultrasound-based pretreatment conditions for *Dictyota dichotoma* biomass to produce reducing sugar was identified as 4.3% (*w*/*v*) algal suspension at 40% amplitude (6.78 MJ/kg) for an incubation of 40 min, resulting in a yield of 0.16 g of the sugars/g of dried biomass [69]. Ngamsirisomsakul et al. [70] demonstrated the influence of glucoamylase supplementation on alkaline-treated *Chlorella* sp. to enhance the yield of reducing sugars and observed an 87% increase (0.281 g/g). Here, the optimum pretreatment condition reported include using 20% of biomass pretreated with 1.5% sulfuric acid, followed by a 20 min incubation at 117 °C. 

The liquid hot water pretreatment of *Scenedesmus* sp. prior to enzymatic saccharification resulted in high glucose recovery under the optimum conditions of a solid-to-liquid ratio, temperature, and incubation period of 1:13 (*w*/*v*), 147 °C, and 40 min, respectively [71]. The addition of glucoamylase and cellulose increased the glucose yield to 14 g/L upon the conversion of the oligosaccharides, with an overall reducing concentration of 0.20 g/g. The glucose recovery was five-fold higher than that in the control (without pretreatment). Bhushan et al. [17] used a crude enzyme from *Chlorella pyrenoidosa* to solubilize microalgae and produce reducing sugars, achieving a yield of 0.19 g/g biomass using 5% (*v*/*v*) of enzyme concentration. During the enzymatic hydrolysis process, the enzymes cellulase, xylanase, and pectinase break the β-1,4 link between the cellulose and hemicellulose, yielding higher sugar concentrations compared to acid hydrolysis. However, the cost ineffectiveness of the enzymatic hydrolysis process hinders its large-scale commercialization, as enzymes are expensive and cannot be recycled due to their denaturation following biomass pretreatment [72]. Moreover, the slow reaction rate of enzymatic hydrolysis impedes the scaling up of biological pretreatment methods. The pretreatment of *Eucheuma spinosum* (red seaweed) with acid (HCl, 0.3 mol/L) for 60 min followed by enzymatic hydrolysis resulted in a high reducing sugar concentration of 21.4 g/L, equivalent to 0.27 g/g of algal biomass [73]. Furthermore, Nordic microalgae, including *Chlorococcum* sp. MC-1, *Desmodesmus* sp. RUC-2, *Coelastrum astroideum* RW-1, and *Chlorella vulgaris* 13-1 biomass grown in wastewater and BG11 media, showed a significant variation in the recovery of total sugar following pretreatment with acid (H_2_SO_4_) and enzymatic hydrolysis [31]. Here, the maximum sugar production reached up to 16.2 g/L. The enzymes secreted by *Aspergillus niger* IB-34 showed high hydrolytic activity towards *Chlamydomonas reinhardtii* biomass [19], achieving a nearly complete saccharification of *Chlorella sorokiniana* and *Scenedesmus obliquus* at a biomass loading of 10% (*w*/*v*), following mild pretreatment at 80 °C for 10 min. Among the various treatments of algal biomass, including acidic, enzymatic, and microwave laser-hydrogen peroxide-Fe-nanoparticle (Mv-H_2_O_2_-Fe) pretreatments, the Mv-H_2_O_2_-Fe pretreatment demonstrated the highest total sugar release of 0.99 g/g of DCW, exceeding the yields of acid and enzymatic treatments, which were 0.59 and 0.49 g/g of DCW, respectively [74]. Furthermore, the Mv-H_2_O_2_-Fe-treated algae exhibited a maximum biopolymer production of 0.74 g/g of DCW. The chemo-enzymatic hydrolysis procedure employed for various microalgae species, including *Tetraselmis striata*, *Tetraselmis* sp., *Cylindrotheca fusiformis*, *Nanofrustulum* sp., *Picochlorum maculatum*, *Phaeodactylum tricomutum*, *Chlorella sorokiniana*, and *Chlamydomonas reinhardtii*, resulted in a maximum reducing sugar production of 34 g/100 g of biomass [75]. Thus, the recovery of sugars from algal biomass is highly influenced by pretreatment approaches. Therefore, selective strategies or combinations of pretreatment methods can be beneficial for the effective valorization of algal biomass for the production of value-added byproducts through fermentation [76,77,78,79,80].

## 3. Production of Polyhydroxyalkanoates from Algal-Biomass-Derived Sugars

Diverse groups of microbes (~200), such as Firmicutes (*Clostridium* and *Bacillus*) and Proteobacteria, produce PHAs [10,81]. The production of PHAs significantly varies among these microbes because of their diverse substrate-utilization efficiencies and the different physiological conditions required for PHA accumulation. PHAs, particularly PHBs, are generally produced as a homopolymer. Moreover, PHA producers utilize the following pathways: (i) the methylmalonyl-CoA pathway, (ii) an all-over-again unsaturated fatty acids engineered pathway, and (iii) a five-step metabolic pathway aided by two stereospecific 2-enoyl-CoA hydratases preceding polymerization [11]. Algal biomass is a suitable low-cost feedstock for producing PHAs and other value-added products through microbial fermentation [82,83,84,85].

Khomlaem et al. [38] evaluated the potential of various bacterial cultures for PHA production, including *Bacillus megaterium* ALA2, *Cupriavidus necator* KCTC 2649, and *H. mediterranei* DSM 1411, using a *Chlorella* sp. biomass hydrolysate. Under batch mode (0.2 L), these bacterial species accumulated PHA in the range of 0.84–7.51 g/L, with content up to 29–75% of their DCW. The PHA content within their biomass showed remarkable variation, which can be attributed to factors such as genetic stability, fast growth, and efficient metabolic activities [38]. Abdelmalek et al. [41] demonstrated the production of PHA using the marine bacteria *Halomonas* spp., *Halomonas pacifica* ASL 10, and *Halomonas salifodiane* ASL 11, cultivated on the hydrolysates of *Spirulina* sp., *Corallina mediterranea*, and *Pterocladia capillacea*. Following an acid pretreatment, the bacteria *H. pacifica* ASL 10 and *H. salifodiane* ASL 11 could accumulate PHA up to 67 and 63% of DCW, respectively. These *Halomonas* spp. accumulated up to 1.2 g/L of PHA from *P. capillacea*, 1.5 g/L of PHA from *Spirulina* sp., and 3.0 g/L of PHA from *C. mediterranea*. The higher accumulation of PHA when using *C. mediterranea* for bacterial growth is primarily attributed to its generation of higher quantities of glucose during acid hydrolysis compared to other algae. Hydrolysates from other algae mainly consist of glucuronic and galactose monomers with polysaccharides containing 4-linked galactose instead of glucose [41]. Bhatia et al. [86] demonstrated PHA production from the red seaweed *Eucheuma spinosum* using various bacterial cultures, including *Bacillus*, *Ralsotonia,* and *Halomonas*. With pure sugars, glucose, galactose, and their mixture as feed, these cultures produced up to 3.02 g DCW/L biomass. However, inhibitory components, such as furfural, hydroxymethylfurfural, and acetate, negatively influenced PHA accumulation in *Halomonas* sp. YLGW01. Biochar derived from *E. spinosum* biomass effectively eliminated up to 88% of phenolics. After phenolic removal, *Halomonas* sp. YLGW01 showed a significant increase in PHA production, reaching 3.88 g/L compared to the control value of 2.58 g/L at 4% NaCl. The PHA content in the biomass increased up to 61.4% of DCW (6.32 g/L). This finding suggests that high PHA production feasibility can be exploited using *E. spinosum*-biomass-derived, inexpensive feed under non-sterile conditions to develop a sustainable and economically viable production system [86]. Wastewater originating from the dairy industry is rich in total sugars, especially lactose, and can effectively support algal growth. Kusmayadi et al. [35] evaluated the dairy wastewater feed-based production of *C. sorokiniana* SU-1 biomass to generate sugar hydrolysate and co-produce PHA and β-carotene using engineered *Rhodotorula glutinis* #100-29. After detoxification, the microalgal hydrolysate yielded a maximum PHA and β-carotene production of 0.90 and 0.09 g/L, respectively. In addition, scaling up this process to a 5 L capacity enhanced the production of PHA (1.83 g/L) and β-carotene (0.13 g/L). Despite the high biomass yield of 11.2 g/L using *C. sorokiniana* SU-1 biomass hydrolysate, a low PHA accumulation of 16.3% of DCW was noted, which could be attributed to the diversion of substrate metabolic flux to other linked metabolisms, i.e., β-carotene as a co-product [35]. *Laminaria japonica* biomass was used as a carbon source to produce PHA using three bacterial isolates: *Paracoccus* sp. LL1, *B. megaterium* ALA2, and *C. necator* NCIMB 11599 [39]. The algal biomass was hydrolyzed using acids to produce 5.9 g/L and 6.1 g/L of reducing sugars when treated with sulphuric acid and hydrochloric acid, respectively. These PHA producers could yield PHA up to 32% of DCW with 2% reducing sugar concentration. Specifically, *C. necator* NCIMB 11599 accumulated 1.58 g/L of PHA. Additionally, in the fed-batch mode of operation of the reactor, the yield of PHA increased to 49% compared to the batch mode yield of 44% [39]. 

Dubey and Mishra [40] studied the efficiency of PHA production using halophilic bacteria grown on glycerol obtained from algal biowaste. Halophilic microbes, including *Halomonas* spp., *H. daqingensis,* and *H. ventosae*, could grow on microalgal biodiesel waste residues with 5% NaCl supplementation. These microbes accumulated up to 0.24 g/L (35% of DCW) of PHA, suggesting that waste biomass can be used efficiently to produce PHA, offering a cost-effective alternative instead of commercially available reagents for PHA production [40]. Ghosh et al. [87] used macroalgae as a nonconventional source of sugar to produce biopolymers. This study explored the growth of *H. mediterranei* and its production of PHA using reducing sugars obtained from different types of macroalgal biomass. Green macroalgae produce more reducing sugars, which were utilized for PHA production. Specifically, when *Ulva* sp. was used to grow *H. mediterranei,* the PHA concentration reached 2.2 g/L (42% of DCW) [87]. Senko et al. [88] explored the ability of *C. necator* B8619 to produce PHA from a co-culture of *Chlorella* sp. and fungal biomass. The mechanical disruption of the cells and the enzymatic hydrolysis of the biomass yielded a reducing sugar concentration of approximately 39.4 g/L, and the conversion rate of the sugars to biopolymers was observed as 0.44 g/L/h. In another study, defatted *Chlorella* biomass was pretreated with sulfuric acid and hydrochloric acids to yield approximately 46 and 52 g/L of reducing sugars, respectively [42]. When *Paracoccus* sp. LL1 was used to convert the reducing sugars to PHA, the hydrolysate from the biomass treated with 0.3N HCl yielded a higher concentration of PHA compared to the other pretreatment procedures. The PHA concentration reached 1.48 g/L accounting for 37.4% of DCW. In addition, a high content of carotenoids (6.08 mL/L) was generated as a co-product during PHA production. Upon scaling up production to 5 L fermenter, a significant increase of 144% (3.62 g/L) and 92% (11.7 mg/L) in PHA and carotenoid production was observed, respectively. Compared to commercial glucose as a sugar source, the PHA yield was 92% higher when microalgal hydrolysate was used to grow *Paracoccus* sp. LL1 [42]. Algal biomass pretreatment with acids or alkalis is generally required to produce PHAs via microbial fermentation. Kargupta et al. [83] demonstrated a greener one-pot method to produce PHA using the bacterium *Saccharophagus degradans* without a pretreatment of brown seaweed. Additionally, the use of a membrane bioreactor resulted in three-fold higher PHA accumulation in batch cultures using seaweed as feed. Azizi et al. [89] demonstrated that *C. nector* PTCC1615 could produce PHB up to 54% of DCW with a yield of 3.93 g/L from brown seaweed (*Sargassum* sp.) hydrolysates derived using acid pretreatment and enzymatic hydrolysis. Additionally, the fermentation of red seaweed *E. spinosum*-derived reducing sugars to PHA by *C. nector* CECT4635 resulted in yields of 0.26 g of PHB/g of reducing sugar and a PHA content of 58% of DCW [73]. Cultures of *Chlorococcum* spp. MC-1, *Desmodesmus* sp. RUC-2, *Coelastrum astroideum* RW-10, and *Chlorella vulgaris* 13-1 biomass-derived sugars resulted in high PHA production by *Halomonas halophila,* yielding up to 1.04, 0.78, 0.08, and 0.05 g/L, respectively [31]. Moreover, the maximum PHA content in the biomass was around 27% of DCW with *Chlorococcum* sp. MC-1-derived sugars. The lower PHA production by other algal biomass hydrolysates may be due to the presence of inhibitory phenolics. However, Nordic-microalgal-derived hydrolysates can be pretreated to detoxify inhibitory compounds and achieve high PHA production [31].

Under phosphate-limiting and low-dissolved-O_2_ (5%) conditions, the fed-batch cultivation of *Halomonas boliviensis* DSM15516 using *G. corneum* hydrolysates resulted in high PHB accumulation and contents of 21.5 g/L and 41% of DCW, respectively [36]. The maximum PHA productivity was 0.46 g/L/h. In addition, a low gluconic acid content (15 g/L) was noted because of the low dissolved O_2_. In another study, *B. megaterium* KCTC 2194 demonstrated a high PHA content of 51.4% of DCW (5.50 g/L) when *Gelidium amansii* hydrolysates were used in batch mode [37]. Furthermore, the strategy of an intermittent feeding of hydrolysate in fed-batch mode enhanced PHA production, resulting in a maximum biomass production and PHA accumulation of 10.1 and 5.50 g/L, respectively. In contrast, the pH-state strategy resulted in a slightly lower biomass production of 8.20 g/L with a PHA content of 53.2% of DCW [37]. A pilot-scale study (40-L) on PHA production by *H. mediterranei* in pneumatically agitated bioreactors (outdoor fermentation) using *Ulva* sp. hydrolysate demonstrated high biomass and PHA productivity of 50.1 and 27.0 mg/L/h, respectively [90]. PHA production reached 56% w/w of biomass with a conversion yield of 0.107 g/g of algal DCW. Using an ultrasonic harvesting approach with energy inputs of 7.8 kWh/m^3^ resulted in a 30% removal efficiency of *H. mediterranei* cells [90]. In another study, the influence of the initial culture density (10–500 g/L) on PHA production by *H. mediterranei* using *Ulva* sp. hydrolysate was evaluated to enhance the conversion yield [91]. Maximum biomass and PHA contents of 56.0 g/L and 49.4%, respectively, were observed at an initial culture density of 50 g/L. Maximum biomass productivity was 0.05 g/L/h, and PHA productivity was 0.024 g/L/h. Remarkably, the productivity and PHA production were similar to those in the standard media. The average molecular weight was approximately 920–960 kDa, with a polydispersity index of approximately 1.6. The primary benefits of using high-cell-density cultivation for PHA production include improved productivity, reduced downstream processing costs, and effective wastewater treatment. Economic analysis predicted *Ulva* sp.’s annual PHA production rate (APPR) cultivated under offshore conditions would be 148 g PHA/m^2^/year. The commercial cost of the PHA ranged from USD 2.4–5.5 per kg. Based on the APPR, the PHA production from *Ulva* sp. could incur an annual income of USD 3369/ha/year [91]. The starch and cellulose extracted fractions of *Ulva* sp. by *H. mediterranei* resulted in lower PHA production yields of 5.1 and 3.5 mg/g dry weight, respectively, compared to the whole biomass yield of 77.8 mg/g dry weight of *Ulva* [92]. Furthermore, economic analysis showed that the direct use of *Ulva* sp. biomass hydrolysate fermentation for PHA is beneficial. In contrast, glucose and hydrochar coproduction from *Ulva* sp. biomass did not provide any remarkable economic benefits [92]. Novel strains *Bacillus pacificus* NAA2 and *Klebsiella quasipneumonia* NAA4 showed a high PHA production of up to 72.7% from seaweed hydrolysate [93]. Details of the algal-biomass-derived sugars used to produce microbial PHAs are presented in Table 1.

## 4. Limitations and Challenges in Microbial Production of Polyhydroxyalkanoates from Algal Biomass

Primarily, sugars used to produce microbial PHAs cover ~50% of the total production cost; therefore, employing inexpensive and renewable feedstocks such as algal biomass can reduce production costs [32,91]. Despite several advantages, the commercialization of microbial PHA production from microalgae-derived sugars has faced numerous challenges [23,31]. A few disadvantages of using algal biomass are low availability due to the insignificant number of algal-based industrial sources, lack of desirable sugars or precursors, and restricted seasonal accessibility of macroalgae [28,85]. One of the main challenges is the high cost of producing microalgae biomass at a large scale, which is often due to the cost of inputs such as nutrients and energy. Additionally, extracting and refining the fermentable sugars from algal biomass for microbial PHA production can be complex and costly [28,31,32]. Still, microbial PHA production is more expensive than petroleum-based plastic [23,85]. To make microalgae-derived microbial PHAs cost-competitive, a multi-dimensional approach, such as an algal biorefinery, may be necessary. Largely, PHB production has been reported by bacteria from algal biomass [31,39,40]. Due to the high crystalline nature and poor mechanical properties of PHB, its application is highly restricted over the broad uses of the copolymers of PHA having desirable properties. Still, achieving the bacterial synthesis of PHA copolymers such as P(3HB-*co*-3HV) from biomass-derived sugars requires precursor supplementation such as valerate in the feed [4,43]. Therefore, the co-digestion of feed containing such precursors and algal biomass can be desirable to synthesize the corresponding copolymers of PHA by microbes [23,43]. While microalgae offer a promising alternative to microbial PHA production, significant challenges must be addressed before their widespread commercialization. The careful consideration of the cost-effectiveness and scalability of the production process, as well as the optimization of the extraction and purification steps, will be crucial in overcoming these challenges and unlocking the full potential of microalgae as a feedstock to produce microbial PHAs [23,32].

## 5. Mechanical and Physiochemical Properties of the Microbially Synthesized PHAs from Algal Biomass

As the most common type of PHA production, PHB has been widely reported from algal biomass through microbial fermentation [23,31]. The broader uses of PHB are restricted because of their stiff and brittle nature with a high degree of crystallinity. Additionally, its melting temperature (T_m_) and decomposition temperature are nearly close to each other at 175 and 185 °C, respectively [85]. The properties of PHAs, such as T_m_, glass transition temperature (T_g_), crystallinity, the modulus of elasticity, breaking strengths, and Mw, are vital for their potential applications. The microbially synthesized P(3HV-*co*-3HV) as a copolymer exhibits lower crystallinity than PHB, resulting in its better mechanical properties, such as Young’s modulus, tensile strength, T_m_, and T_g_ values [23,85]. Therefore, the high degradation potential of copolymers with improved mechanical and physical properties proved beneficial over PHB for various applications [23,43]. *H. mediterranei* and *Ulva* sp.-based-synthesized PHAs showed decomposition and T_m_ of 247 and 176 °C, respectively [91]. Using polystyrene and polymethyl methacrylate standards, the average Mw and Polydispersity Index (PDI) of synthesized PHAs varied in the range of 0.92–0.96 × 106 and 1.56–1.68, respectively. For the polymer to be of varied usage, the average Mw can be high with a low heterogeneity (PDI, 1.5–2.0) [91]. In contrast, PHAs produced by *H. daqingensis* and *H. ventosae* from algal biodiesel waste residues showed higher degradation temperatures of 290–296 °C [40]. The higher degradation temperature can be associated with the presence of slight impurities. The *H. daqingensis*-synthesized PHB showed a low Mw of 0.31 × 10^6^ with a PDI of 1.82 and a T_g_ of 176 °C [40]. PHA synthesized by enriched mixed culture from residual algal biomass exhibited T_g_ and a crystallization temperature of 298 °C [74]. *C. necator* PTCC1615 synthesized PHB showed Tm and crystallinity temperature values of 167 and 99.5 °C, corresponding to standard PHB values of 177 and 88 °C, respectively [89]. The *H. mediterranei*-based copolymers of PHA from *Ulva* sp. hydrolysate exhibited T_m_ and decomposition temperatures of 150–177 and 241–248 °C under various synthesis conditions, respectively [87,90]. Therefore, the physiochemical properties of PHAs, such as thermal stability, mechanical strength, and biodegradability, can be tuned by varying the bacterial strain, fermentation conditions, and the chemical composition of the polymer [32,40,90].

## 6. Genetic Engineering Approaches

PHA production by natural producers is complex owing to the different growth conditions and extended growth periods required to accumulate PHA and its extraction via hydrolysis from their cells [94,95,96]. As a commonly used microbe for recombinant studies, *E. coli* has been extensively used to study PHA production because of its straightforward culture and rapid growth [97]. Incorporating PHA synthesis genes into *E. coli* has enabled efficient and enhanced PHA production at a lower cost compared to other microorganisms. However, studies on recombinant microorganisms that produce PHA from microalgal biomass are limited [80,85]. Developing algal-biomass-based biorefineries presents significant challenges due to seasonal compositional variations, such as carbohydrate content and salinity [98,99]. Jiang et al. [100] produced PHB using microalgal biomass as the substrate. The gene encoding the enzymes involved in the production of PHB by *Massila* sp. (isolated from seaweed) was incorporated into *E. coli* to enhance the PHB yield. A 117% increase in DCW and a 213% increase in PHB production was observed. Sathish et al. [101] used recombinant *E. coli* to treat microalgal biomass and produce PHA, resulting in an accumulation of up to 34% PHB of DCW using pure sugar, which further increased to 51% of DCW when using microalgal biomass as a carbon source for PHA production. Rahman et al. [97] reported that genetically modified *E. colis* secrete PHB directly into the medium, simplifying the downstream processing and extraction of intracellular PHB from bacterial biomass. It was observed that the *PhaP1* encoding phasin, with a lower molecular weight, binds to the PHB molecules, thereby reducing the granule size of the produced PHB and facilitating the secretion of PHB from the cell. In a 5 L fermentation, the engineered *R. glutinis* #100-29 exhibited a high production of PHA and β-carotene up to 1.9 g/L and 4.9 mg/g of reducing sugars from glucose and *C. sorokiniana*-SU-1-derived sugars as carbon sources, respectively [35]. Therefore, the co-production of value-added products during PHA accumulation, such as β-carotene or others, by engineered PHA producers, can benefit the economic viability of bioprocesses. Overall, emerging strategies can prove beneficial for producing PHAs from algal biomass hydrolysates, including designing metabolic pathways for dynamic control over PHA production, engineering salt-tolerant halophilic microbes to improve PHA yields, and the synthetic engineering of microbial consortia for the efficient conversion of diverse algal biomass hydrolysates into desirable copolymers of PHAs [86,98,99].

## 7. Biotechnological Applications of Polyhydroxyalkanoates

**Figure 2:**
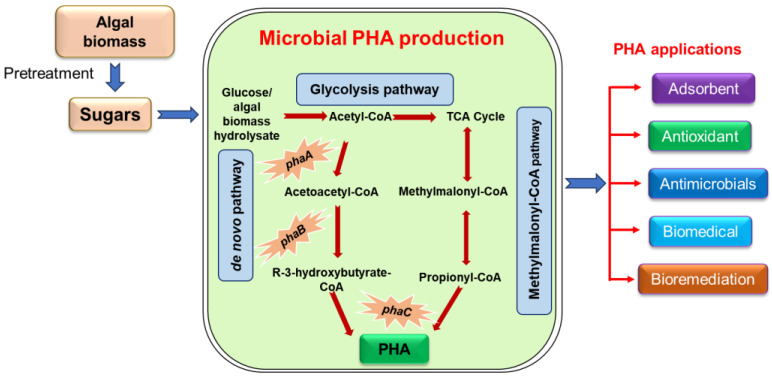
Bacterial conversion of microalgal biomass to PHAs and their biotechnological applications.

The bacterial synthesis of PHA using algal biomass and its biotechnological applications are presented in Figure 2. PHA is a biopolymer that has recently gained significant attention because of its potential applications as an adsorbent [102,103]. PHAs exhibit excellent adsorption properties, making them promising materials for various adsorption applications, such as wastewater treatment, the removal of heavy metals from contaminated water, and the adsorption of organic pollutants [104,105]. Furthermore, their biodegradability and renewability render them environmentally friendly alternatives to conventional adsorbents. The PHA-polyricinoleic acid-Ag nanocomposite exhibited high adsorption efficiency, achieving up to 99.5% removal of methylene blue from wastewater, drinking water, and river water samples [103]. The biodegradable PHA-diethanolamine matrix measured heavy metals such as lead, cadmium, and zinc, with detection limits of 1.05, 0.42, and 0.13 µg/L in food, wastewater, and water samples, respectively [106]. Additionally, the PHA-biochar reactor efficiently adsorbed and biodegraded trichloroethylene [102]. 

PHAs act as potential antioxidants because of their unique properties, which make them highly effective at scavenging free radicals and protecting cells from oxidative damage [107,108]. Poly-lactic acid/PHA film showed a high total antioxidant activity of 36.0 µg Trolox equivalent/cm^2^, which is helpful in effectively preserving pork meat [109]. A significant advantage of using PHAs as antioxidants is their biodegradability, an indication that natural environmental processes can easily break them down, reducing their impact on ecosystems compared with other synthetic antioxidants [110,111]. Furthermore, PHAs can be easily synthesized from renewable carbon sources using metabolic engineering and synthetic biology approaches. This makes them promising alternatives to traditional antioxidants derived from nonrenewable resources, which may harm the environment [109,112,113]. Biodegradable nanoparticulate derived from PHA/diethanolamine-caffeic acid exhibited high antioxidant activity with an inhibitory concentration (IC_50_) of 32.2 mg/L for 2,2-diphenyl-1-picrylhydrazyl (DPPH) scavenging activity and was highly degradable in clay soil within 60 days at 25 °C [111]. In another study, bio-based PHA/tannin films showed remarkable antioxidant activity, achieving 80% scavenging activity within 7 h towards DPPH, making them helpful in food-packaging applications [112]. Multifunctional chromone-incorporated PHA film exhibited antioxidant activity (>55% towards DPPH) and superior antimicrobial activity towards *Aspergillus niger*, *Escherichia coli*, and *Staphylococcus aureus* [114].

Owing to their biocompatibility and bioactivity, these polymers are promising alternatives to conventional antimicrobial agents [115]. PHA/diethanolamine-caffeic acid-based nanoparticles exhibit antimicrobial activity, inhibiting up to 98.0% of food pathogen growth within 48 h of incubation, including *S. aureus* DSM683, *Listeria monocytogenes* DSM19094, *Salmonella enterica* DSM9386, and *E. coli* DSM787 [111]. Similarly, biodegradable bacterial-cellulose-deposited flax fabrics incorporating PHA/polylactic acid showed prolonged antimicrobial activity against *E. coli*, *S. aureus*, and *Pseudomonas aeruginosa* [116]. However, several limitations need to be considered when using PHAs for antimicrobial applications: (i) their limited spectrum of activity, that is, diverse effectiveness against Gram-positive and Gram-negative bacteria and fungi; (ii) the durability of PHAs under different environmental conditions; and (iii) the high cost of PHA production [116,117]. Strategies to overcome these limitations have been proposed, such as modifying the PHA structure, combining PHAs with other antimicrobial agents (nanomaterials), and optimizing production processes to improve activity, durability, and cost-effectiveness [118].

PHAs have gained considerable attention because of their biomedical applications and their biocompatible and biodegradable properties that make them suitable for various medical purposes, such as tissue engineering, drug delivery systems, anticancer activity, and surgical implants [119,120]. PHA-g-cellulose-Fe_3_O_4_/ZnO nanocomposites showed an efficient delivery system for artesunate and dopamine drugs with an IC_50_ of 12.3–21.2 µg/mL, along with robust anticancer activity against HCT-116 cells [118]. Sterilized gauze loaded with 31.1 μg of AgNPs/gelatin/PHA resulted in an effective wound dressing [120]. PHAs offer advantages, such as biocompatibility, controlled degradation, and the ability to be processed into various forms and structures. Their biodegradability ensures that the body can safely break them down over time, thereby reducing the risk of long-term complications [117,120,121]. In addition, the mechanical properties of PHAs can be tailored to meet specific requirements, rendering them versatile for various biomedical applications. Furthermore, PHAs support cell growth and adhesion, making them ideal for tissue engineering applications [3,122,123,124]. Researchers have created drug delivery systems with controlled release capabilities by incorporating bioactive agents such as growth factors or antibiotics into PHA matrices [117,124]. These characteristics make PHAs promising materials for various biomedical applications, offering opportunities for advancements in healthcare and improving patient outcomes [3,120,125]. 

PHAs have shown promising potential in bioremediation owing to their biodegradability and the ability of microorganisms to efficiently degrade various pollutants by utilizing these polymers as a carbon source [126,127]. The use of PHAs in bioremediation can significantly restore polluted environments by providing a sustainable and efficient method for removing contaminants from soil, water, and air [103,104,127]. Furthermore, PHAs offer several advantages for bioremediation applications, including their biodegradability, ability to be utilized as a carbon source and energy reserve by microorganisms, and effectiveness in efficiently degrading various pollutants. Additionally, PHAs can be synthesized from renewable resources, thus reducing the environmental impact of bioremediation efforts [3,103,105]. 

## 8. Perspectives and Concluding Remarks

Microbial PHAs are biodegradable polymers synthesized by various microorganisms for storage under nutrient-limiting conditions. However, their production encounters several challenges: (i) the limited availability of suitable carbon sources for microbial growth and PHA production, (ii) the complexity of regulating and controlling the metabolic pathways involved in PHA synthesis, (iii) the low yield and productivity of PHA production in microbial systems, (iv) the challenges associated with scaling up the production of microbial PHAs to an industrial scale, (v) the need for extensive downstream processing and purification of microbial PHAs, (vi) the potential risk for contamination in microbial PHA production processes, and (vii) the challenges of achieving consistent and reproducible product quality. These challenges hinder the widespread adoption and commercialization of microbial PHAs as sustainable alternatives to traditional petroleum-based plastics. PHAs are highly desirable over homopolymers (PHBs) because of their broad biotechnological applications, especially in the biomedical sector. Generally, precursor supplementation is required during microbial fermentation to produce the corresponding PHA copolymers. Therefore, co-substrate strategies, such as the co-digestion of feed, can be employed to produce desirable copolymers of PHAs with desired variations in their properties. Additionally, the successful conversion of algal biomass into valuable sugars, including PHAs, for various biotransformation applications, is highly dependent on effective pretreatment approaches. Genetically engineered algal cultures show promise for an efficient bioremediation of contaminated wastewater, or GHGs, such as CO_2_ utilization (flue gas as origin), enhancing cell biomass for use as a feedstock for various biotechnological applications, including PHA production. Utilizing algal biomass as a carbon source to produce microbial PHAs on a pilot scale offers a sustainable solution for addressing the issue of plastic waste and reducing dependence on fossil fuels, thereby promoting a circular economy. Furthermore, using genetically engineered PHA for easy recovery or value-added co-product formation can minimize economic competitiveness compared to plastics. Advances in molecular and genetic engineering have enabled the selection of bacterial strains capable of producing PHAs in high quantities, up to 90% of their total biomass. However, the high cost of production remains a significant challenge for the commercialization of PHAs, limiting their widespread adoption.

## Figures and Tables

**Table 1:** Microbial conversion of microalgal-derived biomass hydrolysate into polyhydroxyalkanoates (PHAs).

Algal Biomass	Pretreatment Method	Reducing Sugar (g/L)	PHA			References
			Microbial PHA Producer	Yield (g/L)	Composition	
Algal biodiesel waste	- ^a^	-	*Halomonas daqingensis*	0.24	PHB	[40]
			*Halomonas ventosae*	0.21	PHB	
Algal biomass	Acid and microwave-peroxidenanoparticles	0.99 ^b^	Mixed culture	0.74 ^b^	PHB	[74]
*Chlorella* sp.	Acid (HCl)	20.0	*Bacillus megaterium* ALA2	0.84	PHB	[38]
			*Cupriavidus necator* KCTC 2649	7.51	PHB	
			*Haloferax mediterranei* DSM 1411	3.79	P(3HB-*co*-3HV)	
	Dilute acid (H_2_SO_4_ and HCl)	52.0	*Paracoccus* sp. LL1	3.62	P(3HB-*co*-3HV)	[42]
*Chlorella vulgaris* 13-1	Acid (H_2_SO_4_)	10.8	*Halomonas halophila*	0.05	PHB	[31]
*C. vulgaris* C-1	Acid (H_2_SO_4_ and HCl) and mechanical destruction	39.4	*C. necator* B8619	0.44	PHB	[88]
*Chlorella sorokiniana* SU-1	Acid (H_2_SO_4_)	39.8	*Rhodotorula glutinis* #100-29	1.83	PHB	[35]
*Chlorococcum* sp. MC-1	Acid (H_2_SO_4_)	12.5	*H. halophila*	1.04	PHB	[31]
*Coelastrum astroideu* RW10	Acid (H_2_SO_4_)	11.4	*H. halophila*	0.08	PHB	[31]
*Corallina mediterranea*	Acid (H_2_SO_4_)	-	*Halomonas pacifica* ASL 10	2.80	P(3HB-*co*-3HV)	[41]
			*Halomonas salifodiane* ASL 11	3.00	P(3HB-*co*-3HV)	
*Desmodesmus* sp. RUC-2	Acid (H_2_SO_4_)	16.2	*H. halophila*	0.78	PHB	[31]
*Eucheuma spinosum*	Acid (HCl)	21.4	*C. necator* CECT4635	0.60 ^b^	PHB	[73]
	Dilute acid (H_2_SO_4_)	-	*Halomonas* sp. YLGW01	3.9	PHB	[86]
*Gelidium amansii*	Acid (H_2_SO_4_)	29.1	*Bacillus megaterium* KCTC 2194	5.50	P(3HB-*co*-3HV)	[37]
*Gelidium corneum*	Hydrothermal	-	*Halomonas boliviensis* DSM15516	21.5	PHB	[36]
*Laminaria japonica*	Acid (HCl and H_2_SO_4_)	5.9–6.1	*Paracoccus* sp. LL1	1.58	P(3HB-*co*-3HV)	[39]
		5.8–6.0	*B. megaterium* ALA2	0.65	PHB	
		5.9–6.1	*C. necator* NCIMB 11599	2.39	PHB	
*Pterocladia capillacea*	Acid (H_2_SO_4_)	-	*H. pacifica* ASL 10	1.00	P(3HB-*co*-3HV)	[41]
			*H. salifodiane* ASL 11	1.50	P(3HB-*co*-3HV)	
*Sargassum* sp.	Acid (H_2_SO_4_ and HCl)	-	*C. necator* PTCC1615	3.93	PHB	[89]
*Spirulina* sp.	Acid (H_2_SO_4_)	-	*H. pacifica* ASL 10	1.30	P(3HB-*co*-3HV)	[41]
			*H. salifodiane* ASL 11	1.20	P(3HB-*co*-3HV)	
*Ulva* sp.	Subcritical water	-	*H. mediterranei*	2.20	P(3HB-*co*-3HV)	[87]
				2.08	P(3HB-*co*-3HV)	[90]
				0.12 ^b^	P(3HB-*co*-3HV)	[91]

^a^ Not reported or available; ^b^ yield in g/g of weight or g of PHA/g of DCW.
